# Population-Level Differentiation in Growth Rates and Leaf Traits in Seedlings of the Neotropical Live Oak *Quercus oleoides* Grown under Natural and Manipulated Precipitation Regimes

**DOI:** 10.3389/fpls.2017.00585

**Published:** 2017-05-09

**Authors:** Jose A. Ramírez-Valiente, Alyson Center, Jed P. Sparks, Kimberlee L. Sparks, Julie R. Etterson, Timothy Longwell, George Pilz, Jeannine Cavender-Bares

**Affiliations:** ^1^Department of Ecology, Evolution and Behavior, University of MinnesotaSaint Paul, MN, USA; ^2^Department of Biology, Normandale Community CollegeBloomington, MN, USA; ^3^Department of Ecology and Evolutionary Biology, Cornell UniversityIthaca, NY, USA; ^4^Department of Biology, University of Minnesota DuluthDuluth, MN, USA; ^5^Herbarium Paul C. Standley, Escuela Agricola PanamericanaTegucigalpa, Honduras; ^6^Biltmore Environmental ConsultantsLoveland, CO, USA

**Keywords:** local adaptation, phenotypic plasticity, ecotypes, leaf economics spectrum, *Quercus oleoides*, water stress, neotropics, specific leaf area

## Abstract

Widely distributed species are normally subjected to spatial heterogeneity in environmental conditions. In sessile organisms like plants, adaptive evolution and phenotypic plasticity of key functional traits are the main mechanisms through which species can respond to environmental heterogeneity and climate change. While extended research has been carried out in temperate species in this regard, there is still limited knowledge as to how species from seasonally-dry tropical climates respond to spatial and temporal variation in environmental conditions. In fact, studies of intraspecific genetically-based differences in functional traits are still largely unknown and studies in these ecosystems have largely focused on *in situ* comparisons where environmental and genetic effects cannot be differentiated. In this study, we tested for ecotypic differentiation and phenotypic plasticity in leaf economics spectrum (LES) traits, water use efficiency and growth rates under natural and manipulated precipitation regimes in a common garden experiment where seedlings of eight populations of the neotropical live oak *Quercus oleoides* were established. We also examined the extent to which intraspecific trait variation was associated with plant performance under different water availability. Similar to interspecific patterns among seasonally-dry tropical tree species, live oak populations with long and severe dry seasons had higher leaf nitrogen content and growth rates than mesic populations, which is consistent with a “fast” resource-acquisition strategy aimed to maximize carbon uptake during the wet season. Specific leaf area (SLA) was the best predictor of plant performance, but contrary to expectations, it was negatively associated with relative and absolute growth rates. This observation was partially explained by the negative association between SLA and area-based photosynthetic rates, which is contrary to LES expectations but similar to other recent intraspecific studies on evergreen oaks. Overall, our study shows strong intraspecific differences in functional traits in a tropical oak, *Quercus oleoides*, and suggests that precipitation regime has played an important role in driving adaptive divergence in this widespread species.

## Introduction

Populations of widespread species often experience a broad range of environmental conditions (Marchin et al., 2008; Peguero-Pina et al., 2014). Both adaptation of key functional traits to local environments and phenotypic plasticity are the primary mechanisms by which populations of sessile species, like plants, respond to environmental heterogeneity (Kawecki and Ebert, 2004; Valladareset al., 2006; Savolainen et al., 2007; Pfennig et al., 2010; Matesanz and Valladares, 2014). Studies on patterns of intraspecific genetic variation and phenotypic plasticity in relation to environmental gradients can provide insights into the adaptive significance of traits (Volis et al., 2002; Ghalambor et al., 2007). For example, if a trait is adaptive for a given species, it is expected to show ecotypic differentiation caused by divergent selection pressures, phenotypic plasticity or both (McKay and Latta, 2002; Lucek et al., 2014). Analogously, if a trait is adaptive in a given environment, it is expected to enhance plant performance (e.g., survival, vegetative biomass, seed production) in that environment but not in other environments where the trait is not adaptive (Lande and Arnold, 1983; Dudley, 1996; Etterson, 2004; Donohue et al., 2005).

Leaf traits have been suggested to play a critical role in plant adaptation to the environment. Global scale studies have shown that six leaf traits are critical to explain broad-range patterns of functional variation across biomes: leaf lifespan, specific leaf area (SLA), rates of photosynthesis and respiration and concentrations of nitrogen and phosphorus (Reich et al., 1992, 1997; Wright et al., 2004). A consistent pattern of close coordination and covariation among these traits has been observed, termed the worldwide “leaf economics spectrum” (LES) (Reich et al., 1997, 2007; Wright et al., 2004). The LES represents a continuum of strategies of leaf investment in resource capture and stress tolerance. At one end of the spectrum, resource-acquisition strategies are characterized by leaves that require low structural investment and have high productivity but short leaf life-spans. On the other end are resource-conservative strategies with leaves that require high structural investment and have lower productivity but function for much longer, allowing for sufficient return on investment. Water use efficiency (WUE), which is not included in the LES, is also considered an important trait for resource-use strategies of the plants under dry conditions. WUE is the ratio of photosynthetic carbon gain to the water loss by transpiration. Instantaneous WUE can be estimated using gas exchange measurements, while stable carbon isotope composition can be used to estimate WUE over the period of leaf development and carbon accumulation (Farquhar et al., 1982).

LES traits and water use efficiency reflect resource-use strategies at the whole-plant level. Plants with traits that maximize resource acquisition at the “fast” end of the spectrum are expected to grow quickly while conservative plants at the “slow” end of the spectrum are expected be more tolerant to stress (Westoby et al., 2002; Reich, 2014). Many studies have confirmed that LES traits are associated with plant performance in specific environments (Stanton et al., 2000; Heschel et al., 2002; Etterson, 2004; Heschel and Riginos, 2005; Donovan et al., 2007, 2009). For example, in a study on the annual *Chamaecrista fasciculata*, Etterson (2004) found that SLA was negatively related to seed production in xeric environments but not in mesic environments. She suggested that differences in SLA among populations were the result of adaptation to different precipitation regimes through natural selection. Similar results have been found in long-lived species. For example, in a series of studies on cork oak, Ramírez-Valiente et al. (2010, 2014, 2015a) showed ecotypic divergence in SLA and that trait was negatively associated with aboveground growth in years with long dry seasons. Ramírez-Valiente et al. (2010, 2014) suggested that SLA played an important role for cork oak saplings to adapt to areas with different drought season length and severity. For non LES traits such as WUE, similar studies have been reported (e.g., Dudley, 1996; Heschel et al., 2002). For example, in a study on the annual *Impatiens capensis*, Heschel et al. (2002) showed that genotypes from a xeric population increased WUE in response to dry conditions in a greater extent than genotypes from a mesic population. They also demonstrated that higher WUE conferred fitness advantage under low water availability.

Seasonally-dry tropical ecosystems (SDTE) are characterized by a nearly constant temperature throughout the year and a marked seasonality in precipitation (Murphy et al., 1995; Linares-Palomino et al., 2011). The length of the dry period may vary from a few days to eight months in the most xeric places (Sánchez-Azofeifa et al., 2005; Bond, 2008; Hirota et al., 2011). This spatial variation in precipitation and dry season length exerts contrasting selection pressures that shape species composition and richness in these forests (Ter Steege et al., 2006; Poorter and Markesteijn, 2008; Tomlinson et al., 2013; Vico et al., 2015). As the dry season becomes longer, there is usually an increasing abundance of species with mesomorphic leaves and short leaf life spans which are hypothesized to reduce dry season photosynthetic activity but increase carbon assimilation and growth rates during the wet season when conditions of water availability are more favorable (Cornelissen et al., 1996; Givnish, 2002; Bowman and Prior, 2005; Markesteijn and Poorter, 2009; Vico et al., 2015). This strategy is representative of the “fast” end of the LES spectrum (Reich, 2014). Within species, patterns of functional variation are expected to be similar to broader-scale observations but with a smaller range (Kraft et al., 2008). However, little is known about genetically-based population-level variation in LES traits and water use efficiency in species from SDTE. Studies on intraspecific variation in functional traits for species from these ecosystems have been conducted largely under field conditions, where environmental and genetic effects are confounded (e.g., Choat et al., 2007; Williams et al., 2008). In addition, few studies have explored how LES traits and water use efficiency are related to plant performance in SDTE that vary in precipitation. As a consequence, the adaptive role of these traits in SDTE remains largely unknown.

In this study, we tested for ecotypic differentiation and phenotypic plasticity in LES traits, water use efficiency and growth rates under natural and manipulated precipitation regimes in a common garden experiment where five populations of the neotropical oak *Quercus oleoides* were established. We also examined the extent to which intraspecific trait variation was associated with plant performance and how this relationship varied in response to water availability. Specifically, we ask the following questions:

- Do LES traits, water use efficiency and growth rates respond plastically to water availability in natural and manipulated precipitation regimes?
- Do populations that differ in their climates of origin vary in their trait means and growth rates under common environments?
- Are LES traits and water use efficiency related to plant performance? Does that relationship change in response to water availability in the dry and wet seasons?

We hypothesized, first, that seedlings would exhibit phenotypic plasticity in response to water availability such that carbon assimilation rates, growth rates and SLA would increase while WUE would decrease in treatments with supplemental water in the dry season. Second, we hypothesized that populations would differ in trait means as a result of adaptation to different precipitation regimes. In accordance with interspecific studies in SDTE, we expected that under more xeric environments populations should have evolved toward a “fast” resource-acquisitive strategy to maximize carbon uptake during the wet season by increasing SLA, N, photosynthetic rates and growth rates but decreasing WUE. These trait values would be most pronounced in the wet season and in watering treatments with supplemental water in the dry season (Lambers and Poorter, 1992; Poorter and Garnier, 1999; Westoby et al., 2002; Tomlinson et al., 2013; Reich, 2014; Vico et al., 2015). Finally, we hypothesized that LES traits (SLA, N, photosynthetic rates) would be positively related to seedling growth rates under well-watered conditions (Reich et al., 1997; Poorter and Bongers, 2006; Poorter and Markesteijn, 2008; Wright et al., 2010; Tomlinson et al., 2013). In contrast, we expected that traits related to a “slow” conservative strategy (e.g., WUE) would be positively associated with growth under dry conditions (i.e., in treatments without water supply in the dry season; Dudley, 1996; Heschel et al., 2002).

## Materials and methods

### Study species

*Quercus oleoides* is an interesting study organism for several reasons. First, is widely distributed across a wide range of precipitation regimes (Cavender-Bares et al., 2015). In fact, it spans the largest gradient of dry-season aridity and wet-season rainfall within live oaks (*Quercu*s section *Virentes*, Cavender-Bares et al., 2015) and encompasses one of the highest precipitation ranges within the distribution of the American oaks (Hipp et al, personal communication). Second, differences among populations have been previously reported for growth, photoprotective pigments and leaf traits in seedlings grown under controlled greenhouse and growth chamber conditions in response to cold, light and water stress (Cavender-Bares et al., 2011, 2015; Koehler et al., 2012; Ramírez-Valiente et al., 2015b; Ramírez-Valiente and Cavender-Bares, 2017). Intraspecific differences in germination (Center et al., 2016) and survival (Deacon and Cavender-Bares, 2015) have also been observed in common gardens in the field.

### Study site and common garden

**Figure 1:**
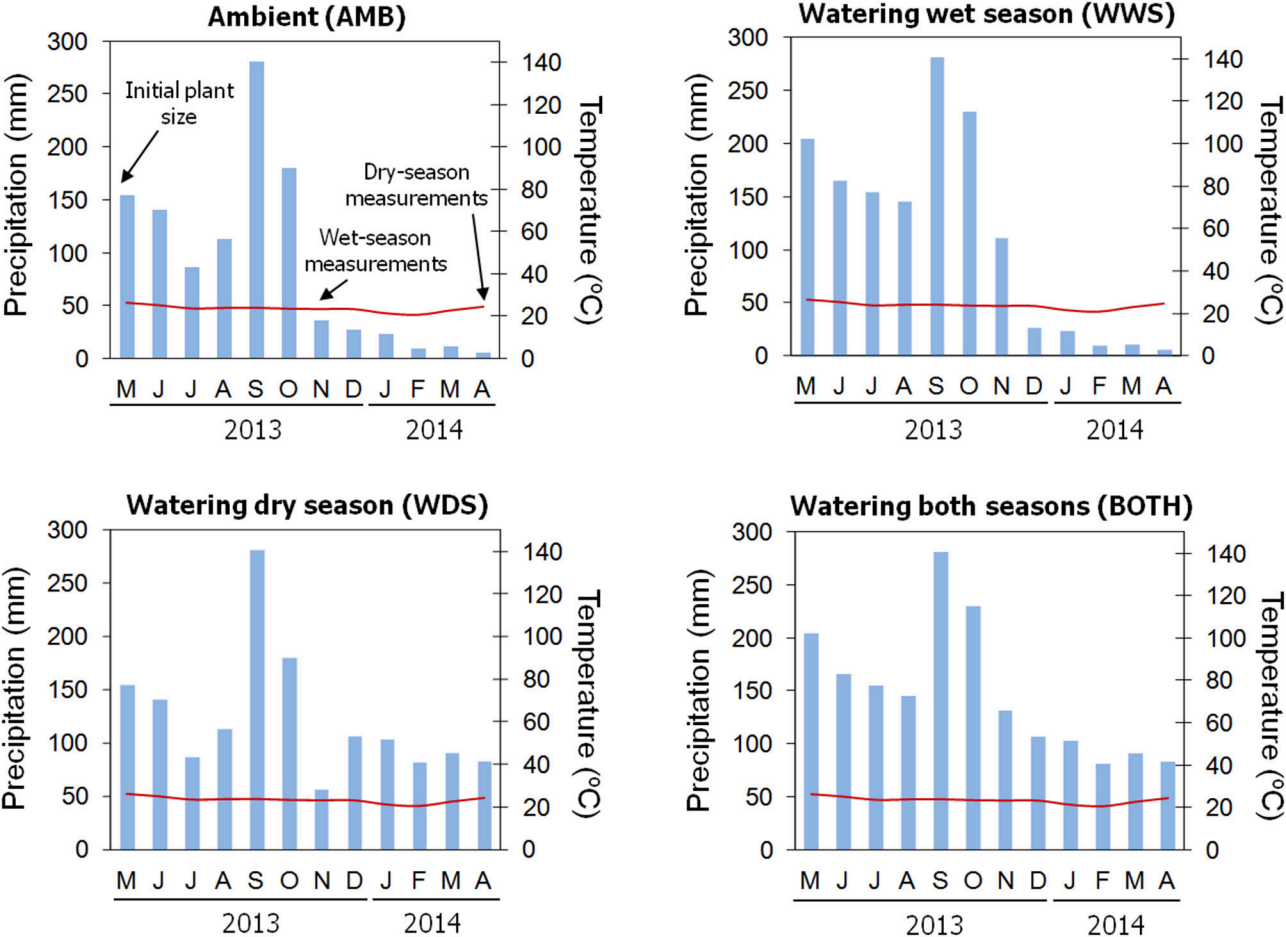
**Representation of the average monthly rainfall (blue bars) and temperature (red line) for the four precipitation regimes implemented in the common garden trial**. Ambient values were obtained from the meteorological station located at the common garden. Estimated total precipitation per month was calculated based on the amount of water added in each watering treatment (see Materials and Methods). The time when growth and morpho-functional measurements were taken is indicated by arrows in the “AMB” figure.

In 2010, we collected acorns from 19 populations belonging to five climatically contrasting regions where *Quercus oleoides* is present (Belize, Honduras, Mexico, dry Costa Rica and mesic Costa Rica). Seeds were stored at 4°C until sown in shadehouses. Eighty one maternal families from eight populations (between 2 and 35 maternal families per population) were finally available for the experiment due to low germination rates (Table 1). After 6 months of growth, seedlings were transferred to a common garden trial in the field in January 2011 within Zamorano University campus (14° 00′ N, 87° 00′ W, 797m, Francisco Morazán, Honduras). This area is characterized by a seasonally-dry tropical climate with mostly constant temperatures throughout the year but a marked seasonality in precipitation (Figure 1). It is located in the driest region of the entire *Q. oleoides* range with up to 6 months of length for the dry season (Figure 1, see Cavender-Bares et al., 2015 for more information). The climate of this area is also characterized by a little dry season in the middle of the wet season locally known as “veranillo.” This is a 2-month period (July and August) during which precipitation may be below potential evapotranspiration although its duration and intensity is highly variable across years (Cavender-Bares et al., 2015).

**Table 1:** **Location sites and climatic characterization of the studied populations and their belonging to climatic regions**.

**Region**	**Population**	**Latitude**	**Longitude**	**Altitude**	**T**	**P**	***I*_m_**
BZ	Hattieville Bypass	17° 34′ 39″ N	88° 22′ 17″ W	6	25.3	1,948	323.1
BZ	Buttercup Estates	17° 33′ 09″ N	88° 24′ 51″ W	12	25.2	2,067	444.9
BZ	Hattieville—Northern Highway	17° 33′ 06″ N	88° 24′ 46″ W	12	25.2	2,067	444.9
BZ	Hattieville	17° 28′ 45″ N	88° 24′ 21″ W	8	25.4	2,079	451.8
HN	Chagüite Maraita	13° 55′ 41″ N	87° 00′ 41″ W	1,017	21.7	1,044	−467.5
HN	Sabana Grande	13° 49′ 08″ N	87° 14′ 50″ W	1,113	21.1	1,170	−325.7
DCR	Santa Elena	10° 55′ 08″ N	85° 36′ 44″ W	282	24.8	1,749	143.0
MCR	Guachipelin	10° 43′ 09″ N	85° 24′ 54″ W	550	23.4	2,283	722.3

*BZ, Belize; HN, Honduras; DCR, Dry Costa Rica; MCR, Mesic Costa Rica. Altitude is given in meters a.s.l., T is annual mean temperature (°C), P is annual precipitation (mm) and I_m_ is an index of moisture (see Materials and Methods for details)*.

The common garden experiment followed a randomized block design with 24 blocks. Because of low germination rates, maternal families were not evenly represented across blocks and the resulting design was unbalanced. Seedlings were planted 50 cm apart, which minimized competition and shading. Four watering treatments (applied to six blocks each) were implemented 2 weeks after transplanting: (1) Ambient rainfall (no water added, hereafter, AMB); (2) Water added in the amount of 18 mm/week during the dry season (hereafter referred to as WDS), (3) Water added up to 25 mm/week during the wet season if it rained less than 25 mm/week (hereafter referred to as WWS), (4) Water added during both the dry and wet seasons following WDS and WWS protocols (hereafter BOTH). These treatments are representative of the natural precipitation gradient where *Q. oleoides* can be found. Blocks were weeded biweekly to reduce competition effects.

### Growth and trait measurements

In May 2013, during the onset of the wet season (Figure 1), initial growth measurements were taken on all plants in the common garden (*N* = 634 plants). Stem height, basal diameter and number of leaves were recorded. The same process was repeated in November 2013 and March–April 2014 (Figure 1). Growth that occurred between May 2013 and November 2013 represents wet season growth, and growth between November 2013 and April 2014 represents growth during the subsequent dry season (Figure 1). Wet and dry season growth was estimated using three separate metrics: height, basal diameter and number of leaves. Metrics of growth were transformed into total dry biomass values using an allometric equation: Dry mass (g) = 0.5018*x* + 0.1540*y* + 0.3209*z, R*^2^ = 0.93, *P* < 0.0001, where *x* is the height (cm), *y* is the diameter (mm) and *z* is the number of leaves. The allometric equation was constructed using 120 harvested plants up to 120 cm tall established in a greenhouse. A multiple regression was performed including total biomass as the dependent variable and height, diameter and number of leaves as dependent variables (See Cavender-Bares et al., 2004 for a similar procedure). Absolute growth rate (g day^−1^) was calculated in each season as AGR = (M_final_ − M_initial_)/(T_final_ − T_initial_), where M_final_ is the estimated biomass at the end of the season, M_initial_ is the estimated biomass at the beginning of the season, T_final_ is the date of the measurement at the end of the season and T_initial_ is the date at the beginning of the season. Relative growth rate (g g^−1^ day^−1^) was calculated as RGR = [log_*e*_(M_final_) − log_*e*_(M_initial_)]/(T_final_ − T_initial_).

In November 2013 (wet season) and April 2014 (dry season), one mature but recently expanded sun-exposed leaf was selected from each plant for gas exchange measurements. Gas exchange measurements were taken with LI-COR 6400 (LI-COR, Lincoln, NE, USA) from 8:30 a.m. to 12:00 p.m. in ten and nine consecutive sunny days in the wet and dry seasons, respectively. Photosynthetic rate (A) and stomatal conductance (g_s_) were measured at ambient atmospheric [CO_2_] ~400 μL L^−1^ and relative humidity (~50%) with saturating light controlled at 1,500 μmol m^−2^ s^−1^ (10% blue light), block temperature set at 25°C, with vapor pressure deficit (VPD) varying between 1.51 and 3.39 in the chamber. Intrinsic water use efficiency (WUEi) was calculated as A/g_s_. Leaves were collected and pressed in labeled coin envelopes. The petiole was removed and fresh leaves were scanned to obtain lamina area and then dried at 65°C. Specific leaf area was calculated as leaf area/dry weight and used to estimate photosynthetic rate and stomatal conductance on a mass basis. In a sub-sample of 7–9 randomly selected plants per block, we measured water potential at predawn (ψ_pd_) using a Scholander pressure chamber (Soil Moisture Equipment Corp., Santa Barbara, CA, USA) in November 2013 (wet season) and April 2014 (dry season) at the same dates as gas exchange measurements.

In April 2014 (dry season), we measured three additional traits in the selected leaves: leaf lamina thickness, Nitrogen concentration (N_mass_) and stable carbon isotope composition (δ^13^C). δ^13^C is a surrogate of integrated water use efficiency during the time for which the plant has fixed carbon (Farquhar et al., 1982; Farquhar and Richards, 1984). Leaf lamina thickness was measured in fresh leaves using a micrometer. Once leaves were dried, nitrogen concentration (N_mass_) and stable carbon isotope composition (δ^13^C) were estimated using a mass spectrometer (Cornell Stable Isotope Laboratory, NY, USA).

### Statistical analyses

Mixed models included population, season, watering treatment, and their interactions as fixed factors. Maternal family nested within population, and block nested within watering treatment were included as random factors. Initial plant biomass was included as a covariate. Three-way interactions were not significant for any trait and removed from final models (data not shown). Since A and g_s_ are non-linearly related, WUEi (A/g_s_) might vary intrinsically with g_s_. Prior to performing mixed models for WUEi, we tested the effect of g_s_ on WUEi and the interaction between fixed factors and g_s_ on WUEi; we plotted g_s_ vs. A for each combination of region/treatment/season and fitted the data to hyperboles (Figure S1). Then, we calculated WUEi at three fixed g_s_ values (0.1, 0.2, 0.3) that covered most of the range of variation of g_s_ in our dataset. We used region rather than population for this test because the small number of plants within some populations did not allow us to fit hyperbolic functions accurately. Our results did not reveal any interaction between g_*s*_ and region, season, or treatment on WUEi (Table S1), indicating that the effect of g_s_ on WUEi did not differ among levels of the three fixed factors. Consequently, individual values of WUEi were used for subsequent analyses.

In order to test whether growth and functional traits were associated to climate of origin; when differences among populations were found, we performed linear regressions between an index of moisture and traits. The index of moisture (*I*_m_) was calculated as *I*_m_ = ∑(*P*_*i*_ − *PET*_*i*_), where *P*_*i*_ is the monthly precipitation and *PET*_*i*_ monthly potential evapotranspiration (see Ramírez-Valiente and Cavender-Bares, 2017 for more details).

We also tested associations between plant performance and functional traits by analyzing trait-growth relationships using semi-partial correlations. This was done by performing Pearson correlations between the residuals of AGR and RGR obtained from mixed models and individual trait values. Relationships between traits and survival were not explored because only 14 plants died during the studied period (all of them in the wet season). Traits measured in both seasons that showed significant relationships with growth rates were used for further analyses. We then conducted two mixed-models including AGR or RGR as dependent variables and individual traits as covariates. Population, season, and treatment were included as fixed factors. Maternal family nested within population, and block nested within watering treatment were included as random factors. Initial plant biomass was included as a covariate. In order to avoid colinearity, we excluded mass-based photosynthesis and stomatal conductance because of their high correlation with area-based measurements. Results were very similar when mass-based rather than area-based measurements were included (data not shown). We also included the interactions of *season by “trait”* that would indicate a significant difference in the relationship between the trait and growth across seasons. Interactions of *treatment by* “*trait”* were not included in these analyses due to the absence of treatment effect for leaf traits. Interactions of *populations by* “*trait”* were not significant for any trait and were removed from final models. We also explored relationships between traits by performing Pearson correlations. Statistical analyses were performed using packages lme4 and lmerTest in R 3.2.2 (R Core Team, 2015) and STATISTICA 10.0 (StatSoft, Inc., Tulsa, OK, USA). Sigmaplot 12.5 (Systat Software Inc., San Jose, CA, USA) was used to plot some figures.

## Results

**Figure 2:**
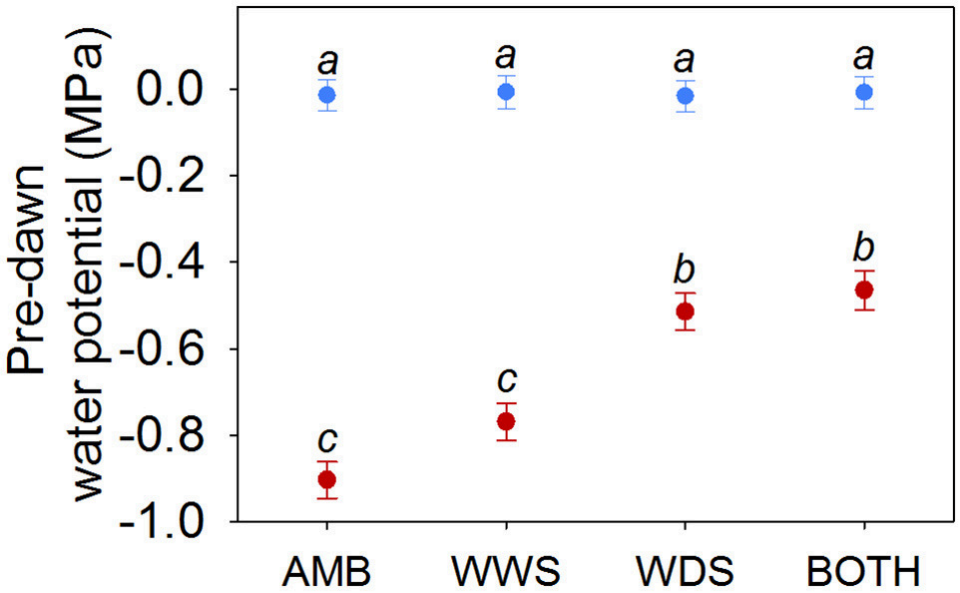
**Least-squares means (±SE) for pre-dawn water potential (MPa) in the dry season (red) and wet season (blue)**. AMB, ambient; WWS, watering in the wet season; WDS, watering dry season; BOTH, watering in both seasons. Homogeneous groups at the 95% confidence level are presented by the same letter for *treatment by season* effect.

There was a significant season by treatment interaction for pre-dawn water potential ($\chi_{3}^{2}$ = 31.47, *P* < 0.001). BOTH and WDS treatments (the two where plants received supplemental water in the dry season) had higher ψ_pd_ values than plants established in AMB and WWS treatments (the two where plants did not receive supplemental water during the dry season) (Figure 2). The supplemental water supply in the dry season was intended to match that of the most mesic dry season conditions in the species range but not to simulate the wet season; as a consequence, plants in BOTH and WDS treatments had also more negative ψ_pd_ in the dry season than in the wet season, and season had a strong effect on ψ_pd_ regardless of watering treatment ($\chi_{1}^{2}$ = 2807.45, *P* < 0.001, Figure 2).

**Figure 3:**
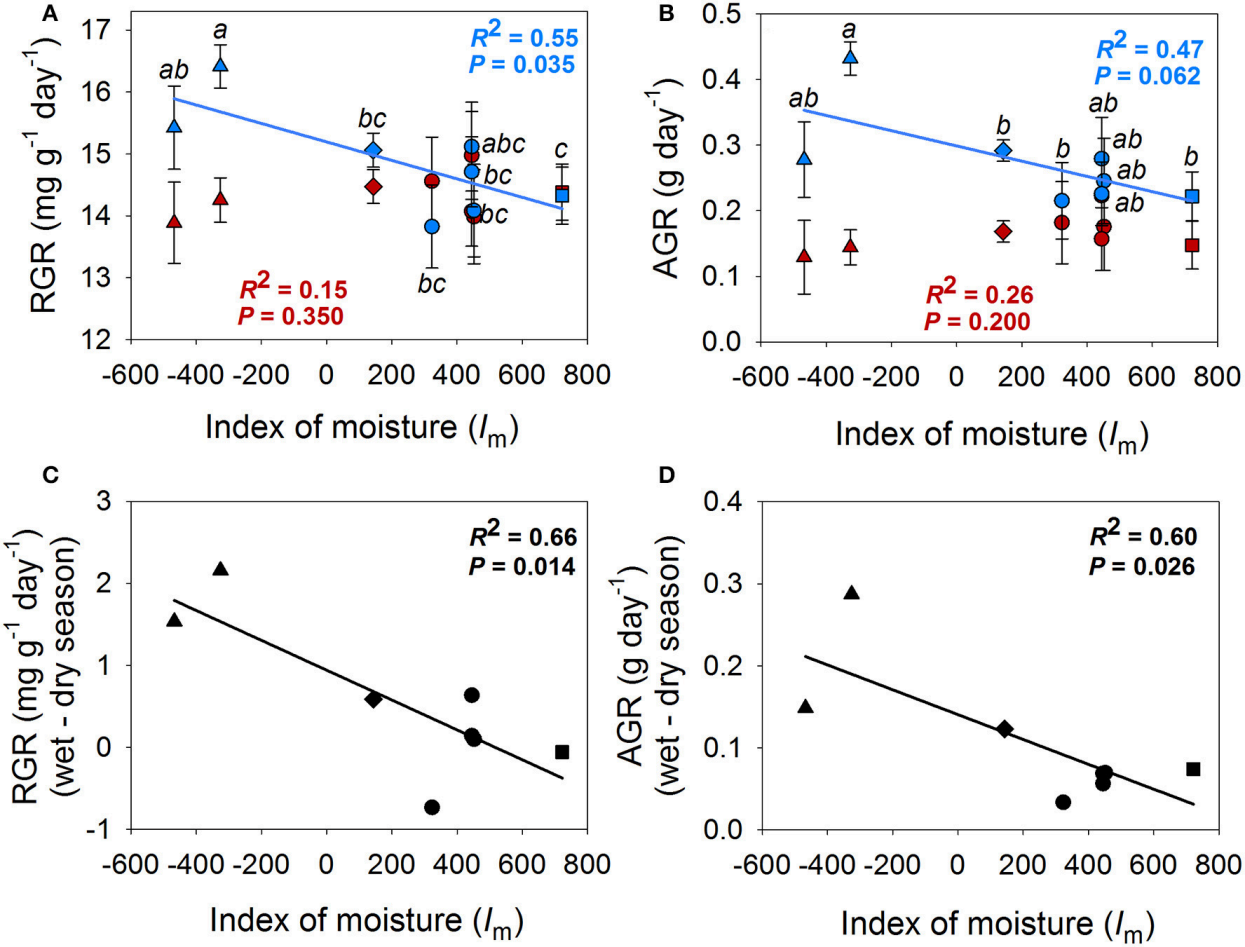
**Population means for relative growth rate (RGR) (A)**, absolute growt rate (AGR) **(B)** in the wet season (blue) and dry season (red) in realtion to the index of moisture of the population. **(C)** and **(D)** show the relationships between the difference in RGR and AGR between seasons and the index of moisture of the population (*I*_m_). Points indicate population means. Standard errors could only be calculated for intra-season means **(A,B)**. Populations that belong to the same climate region are represented by the same symbols; Honduras: triangles, Dry Costa Rica: diamonds, Belize: circles, Mesic Costa Rica: squares.

Mixed models showed differences between seasons for all traits measured in both seasons (Tables 2, 3). Plants exhibited larger growth rates (RGR and AGR), SLA, photosynthetic rates (A_area_, A_mass_) and stomatal conductance (g_s, area_, g_s, mass_) in the wet season and had higher values of intrisic water use efficiency (WUEi) in the dry season (Figure 3, Figure S2).

**Table 2:** **Results of mixed models for relative growth rate (RGR), absolute growth rate (AGR), specific leaf area (SLA), leaf thickness (Thickness), mass-based leaf nitrogen content (N_**mass**_) and 13-Carbon isotope composition (δ^**13**^C)**.

		**RGR**		**AGR**		**SLA**		**Thickness**		**N_mass_**		**δ^13^C**	
**Effect**	**df**	χ^2^		χ^2^		χ^2^		χ^2^		χ^2^		χ^2^	
Population	7	**25.24**	^***^	**21.19**	^**^	**56.93**	^***^	**28.26**	^***^	**32.18**	^***^	4.136	
Season	1	**23.31**	^***^	**93.16**	^***^	**168.2**	^***^	−		−		−	
Treatment	3	1.923		1.734		0.387		2.383		1.437		0.411	
Population × Season	7	**25.29**	^***^	**28.51**	^***^	3.693		−		−		−	
Population × Treatment	21	**39.53**	^**^	12.27		25.21		15.20		6.498		16.84	
Season × Treatment	3	3.884		1.437		**11.14**	^*^	−		−		−	
Block (Treatment)	1	**102.7**	^***^	**22.62**	^***^	**178.1**	^***^	**66.05**	^***^	**52.92**	^***^	**82.74**	^***^
Family (Population)	1	0.766		0		2.84		**10.88**	^***^	**5.216**	^*^	0.964	
Initial size	1	**262.2**	^***^	**90.83**	^***^	**27.10**	^***^	**85.16**	^***^	2.701		**16.56**	^***^

*Population, season, treatment and their interactions are fixed factors. Initial size is a covariate and block and maternal family are random factors. Degrees of Freedom (df), chi-square values (χ^2^) and significance P values were obtained from likelihood-ratio tests comparing reduced models to the full model using function “anova” in lme4 package*.

* P < 0.05,

** P < 0.01,

*** *P < 0.001. Significance effects (P < 0.05) are typed in bold. Thickness, N_mass_, and δ^13^C were measured only in the dry season*.

**Table 3:** **Mixed model results for area-based photosynthesis (A_**area**_), mass-based photosynthesis (A_**mass**_), area-based stomatal conductance (g_**s, area**_), mass-based stomatal conductance (g_**s, mass**_), instantaneous water use efficiency (WUEi)**.

		**A**_**area**_		**A**_**mass**_		**g**_**s, area**_		**g**_**s, mass**_		**WUEi**	
**Effect**	**df**	χ^2^		χ^2^		χ^2^		χ^2^		χ^2^	
Population	7	2.519		11.64		2.800		**15.08**	^*^	4.280	
Season	1	**17.43**	^***^	**111.8**	^***^	**146.5**	^***^	**276.9**	^***^	**168.5**	^***^
Treatment	3	3.332		3.357		**13.00**	^**^	5.590		**9.367**	^*^
Population × Season	7	2.212		3.772		2.602		4.598		7.147	
Population × Treatment	21	25.76		17.48		16.16		12.68		10.02	
Season × Treatment	3	**10.39**	^*^	**14.02**	^**^	**28.36**	^***^	**30.37**	^***^	**59.21**	^***^
Block (Treatment)	1	**107.7**	^***^	**79.45**	^***^	**35.91**	^***^	**76.30**	^***^	**61.34**	^***^
Family (Population)	1	**8.46**	^**^	**11.27**	^***^	**5.680**	^*^	**6.780**	^**^	0	
Initial size	1	0.067		3.084		**13.27**	^***^	**17.67**	^***^	**45.78**	^***^

*Population, season, treatment and their interactions are fixed factors. Initial size is a covariate and block and maternal family are random factors. Degrees of freedom (df), chi-square values (χ^2^) and significance P values were obtained from likelihood-ratio tests comparing reduced models to the full model using function “anova” in lme4 package*.

* P < 0.05,

** P < 0.01,

*** *P < 0.001. Significance effects (P < 0.05) are typed in bold*.

There was also a “*season by treatment”* interaction for SLA and gas exchange traits (Tables 2, 3). Specifically, plants tended to have higher values of SLA, photosynthetic rates (A_area_, A_mass_) and stomatal conductance (g_s, area_, g_s, mass_) and lower values of WUEi in WDS and BOTH than AMB and WWS treatments in the dry season (Figure S2). No differences among treatments were found for leaf thickness, nitrogen concentration (N_mass_) and stable carbon isotope composition (δ^13^C) measured in the dry season (Table 2).

**Figure 4:**
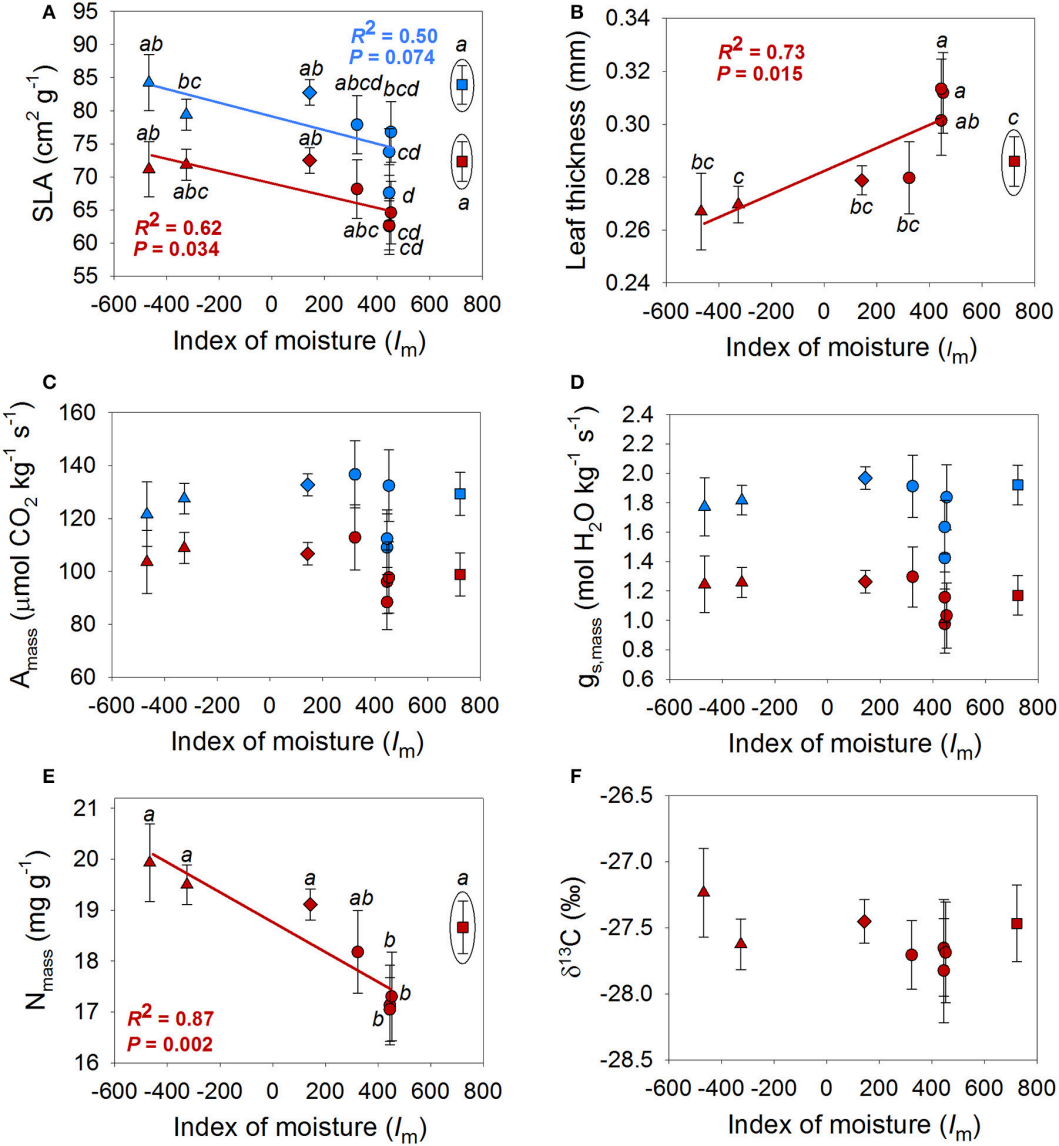
**Population means for specific leaf area (SLA) (A)**, leaf thickness **(B)**, mass-based photosynthetic rate (A_mass_) **(C)**, mass-based stomatal conductance (g_s, mass_) **(D)**, mass-based leaf nitrogen content (N_mass_) **(E)** and 13-Carbon isotope composition (δ^13^C) **(F)** in the wet season (blue) and dry season (red) in relation to the index of moisture of the population (*I*_m_). Populations that belong to the same climatic region are represented by the same symbols are; Honduras, triangles; Dry Costa Rica, diamonds; Belize, circles; Mesic Costa Rica, squares. Linear fits (*R*^2^) and significant levels (*P*) were obtained when the “Mesic Costa Rica” population was excluded from the analyses (see further explanation in the text). Only significant or marginally significant relationships are shown.

Mixed models revealed differences among populations in AGR, RGR, SLA, leaf thickness, N_mass_ and g_s, mass_ (Tables 2, 3 and Figures 3, 4). A population by season interaction was also significant for AGR and RGR (Table 2). There was a negative relationship between the index of moisture of the maternal source (*I*_m_) and growth rates in the wet season (Figure 3). There was also a negative relationship between *I*_m_ and N_mass_ (*R*^2^ = 0.55, *P* = 0.036, Figure 4), and a marginal positive relationship between *I*_m_ and leaf thickness (*R*^2^ = 0.49, *P* = 0.053, Figure 4). The rest of the traits did not show any association with the climate of the source (*P*-values and *R*^2^ coefficients for non-significant relationships are not shown). However, once the mesic population from Costa Rica was excluded from the analyses, the relationships between trait and climate became much stronger for SLA, leaf thickness and N_mass_ (Figure 4).

In the dry season, A_area_, leaf thickness and δ^13^C were positively associated with RGR while SLA was negatively associated with RGR (Table 4). In the wet season, A_area_, A_mass_ and WUEi were positively associated with RGR, while SLA was negatively associated with RGR (Table 4). Results for AGR were very similar to those reported for RGR (Table 4). “Multiple-trait” mixed models that accounted for trait correlations showed that SLA and A_area_ were the best predictors of growth rates (Table S2). Mixed models also revealed a significant *season by SLA* interaction for AGR, indicating that SLA had a stronger relationship with AGR in the wet season than in the dry season (Table S2).

**Table 4:** **Semi-partial correlations between traits and residuals of RGR (relative growth rate) and AGR (absolute growth rate) obtained from mixed models**.

	**RGR**		**AGR**	
	**Dry season**	**Wet season**	**Dry season**	**Wet season**
SLA	−**0.154**	−**0.173**	−0.050	−**0.111**
A_area_	**0.103**	**0.216**	**0.090**	**0.153**
A_mass_	0.028	**0.105**	0.070	0.072
g_s, area_	0.034	0.049	0.039	0.012
g_s, mass_	−0.025	−0.047	0.023	−0.051
WUEi	0.072	**0.184**	0.022	**0.154**
Thickness^a^	**0.170**	–	**0.122**	–
${\text{N}}_{\text{mass}}^{\text{a}}$	−0.002	–	−0.018	–
δ^13^C^a^	**0.109**	–	0.079	–

*SLA, Specific Leaf Area; A_area_, area-based photosynthesis; A_mass_, mass-based photosynthesis; g_s, area_, area-based stomatal conductance; g_s, mass_, mass-based stomatal conductance; WUEi, intrinsic water use efficiency, Leaf thickness; N_mass_, mass-based nitrogen content; δ^13^C, carbon-13 isotope composition (δ^13^C). Significant correlations (P < 0.05) are given in bold*.

a *Measured only in the dry season*.

## Discussion

In this study, we tested for population differentiation and phenotypic plasticity of leaf economics traits, water use efficiency and growth rates in seedlings of *Quercus oleoides* grown under natural and manipulated precipitation regimes in a common garden in southern Honduras. Our results revealed phenotypic plasticity in most of these traits. Seedlings growing in treatments with low water availability in the dry season showed reduced carbon assimilation rates, growth rates, SLA, and increased water use efficiency. Consistent with our expectations, populations differed in growth rates, SLA, N_mass_ and leaf thickness. Populations with long and severe dry seasons had higher growth rates than mesic populations consistent with a “fast” resource-acquisition strategy. SLA was the best predictor of plant performance, but contrary to expectations it was negatively associated with relative and absolute growth rates in both wet and dry seasons.

### Phenotypic plasticity and population differences in growth rates, LES traits and water use efficiency

All traits measured in both dry and wet seasons exhibited high phenotypic plasticity to seasonal precipitation (question i, Figures 2–4). As observed in other oak species from seasonally-dry regions, growth rates, gas exchange and SLA showed highly plastic responses to temporal variation in precipitation (Ramírez-Valiente et al., 2010, 2014, 2015a). These traits also showed plastic responses to watering treatment (measured mainly by treatment-by-season effect) but to a lesser extent than season itself (Figure S2). This pattern indicates that differences in water input to soils during the dry season elicits plastic trait and growth responses, despite high evaporative demand and high VPD values.

Most importantly, our study revealed remarkable differences among populations of *Quercus oleoides* in growth rates, leaf morphology, N_mass_ and g_s_ (question ii). Wet season growth rates were negatively associated with *I*_m_ in the source populations (Figure 3). In other words, populations from the more xeric sites had higher growth rates under favorable conditions in the wet season than the more mesic populations. These results are in agreement with comparative species-level studies in seasonally-dry tropical ecosystems, which tend to show that species from xeric regions (long and severe dry seasons) evolve toward a resource-acquisition strategy with increased growth potential during the favorable season (Cornelissen et al., 1996; Givnish, 2002; Bowman and Prior, 2005; Markesteijn and Poorter, 2009; Tomlinson et al., 2013). Interestingly, these results contrast those found for evergreen oak species from seasonally-dry temperate zones, which show that populations from xeric climates with long dry seasons have reduced growth potential (Gratani et al., 2003; Ramírez-Valiente et al., 2010, 2014; Niinemets, 2015). These differences are probably explained by the fact that photosynthetic activity is limited by both water deficit in summer and cold temperatures in winter in Mediterranean-type ecosystems (Larcher, 2000; Nardini et al., 2000; Cavender-Bares et al., 2005; Flexas et al., 2014; Granda et al., 2014; Niinemets, 2016). In contrast to Mediterranean ecosystems, which have cold winters, in seasonally-dry tropical ecosystems, temperature is not a limiting factor for photosynthesis in the wet season. In Mediterranean areas where the dry season is long and winters are cold, species tend to have conservative resource-use strategies (long leaf life spans, thick leaves, high density tissues and high water use efficiency) allowing them to reach a positive carbon balance over the course of the year. In contrast, in seasonally-dry tropical ecosystems where the dry season is long, a resource-acquisition strategy that maximizes growth in the warm wet season is expected to be beneficial (Reich et al., 1997; Givnish, 2002; Villar et al., 2005; Markesteijn and Poorter, 2009; Vico et al., 2015).

Leaf economics spectrum traits (SLA, leaf thickness, N_mass_, A_mass_, g_s, mass_) also showed significant differences among populations (Figure 4). Consistent with a “fast” resource-acquisition strategy, which we hypothesized would evolve in more xeric populations with longer dry seasons, N_mass_ and SLA were negatively related to *I*_m_ while leaf thickness was positively related to *I*_m._ Similar patterns have been observed for growth rates in species from SDTE (Markesteijn and Poorter, 2009; Tomlinson et al., 2013). However, the patterns of trait variation in relation to *I*_m_ were less clear than in our previous greenhouse experiment with five *Q. oleoides* populations from the southern distribution of the species range (Figure 4, Ramírez-Valiente and Cavender-Bares, 2017). One possible explanation for the weaker associations between trait variation and climate found in the present study may result from interaction with biotic factors, such as herbivory or pathogens in the field experiment. Alternatively, or in addition, contrasting abiotic environmental variables in the field compared to the greenhouse, such as higher light levels and less variable daylengths, more variable vapor pressure deficits, more extreme soil drought and moisture conditions, or the absence of pot restrictions could have triggered differences in the expression of leaf traits between environments. For example, Conner et al. (2003) observed an increased expression of the genetic variance (i.e., greater differences among genotypes) for floral traits of the wild radish *Raphanus raphanistrum* in a greenhouse compared to the field. They suggested that this observation could be caused by a few specific genotypes with different trait expression across environments (Roles and Conner, 2008). In our previous experiment, a mesic population from Costa Rica had the lowest values of SLA, the smallest leaves and the highest leaf thickness, contrary to the findings obtained in this study. In fact, after removing that population from analyses, the relationships between SLA, leaf thickness and N_mass_ became significant or marginally significant and similar to the patterns observed in the greenhouse (Figure 4). These findings suggest a steep reaction norm between the greenhouse and the field for this population (i.e., genotype by environment interaction) compared to the other studied populations. Interestingly, plants from the mesic higher elevation site in Costa Rica, exhibit neutral genetic differentiation relative to lowland (dry) populations of *Q. oleoides* from Costa Rica (Deacon and Cavender-Bares, 2015). Further research is needed to determine the mechanisms causing this contrasting expression of leaf traits between experiments, particularly for the Costa Rican populations, which have been genetically isolated from the rest of the species (Cavender-Bares et al., 2011, 2015).

Populations did not differ in either instantaneous (measured by gas exchange) or integrated (measured by δ^13^C) water use efficiency. These results are contrary to our expectations for a higher WUE as part of a resource-conservative strategy in populations from short dry seasons. One possibility to explain this unexpected result could be that *Q. oleoides* responds to water stress by using other traits rather than increasing WUE. In fact, populations with short dry seasons did have other traits associated with a resource-conservative strategy such as low SLA and high leaf thickness, which should reduce water loss and conserve water for the plant over longer periods (Dudley, 1996; Lamont et al., 2002; Etterson, 2004). Similarly, in our previous study, we observed that populations of *Q. oleoides* did not vary in WUE but did differ in water potential at the turgor loss point (π_tlp_) under dry conditions (Ramírez-Valiente and Cavender-Bares, 2017). In other words, all populations have similar WUE but populations from short dry seasons had lower π_tlp_ (i.e., increased drought tolerance) in response to drought. On the other hand, it is worth mentioning that when a given species is the subject of study, genetically-based population differences are not always consistent across studies. For example, in Mediterranean evergreen oaks, Gimeno et al. (2009) and Ramírez-Valiente et al. (2011, 2014) did not find population-level variation in (instantaneous or integrated) WUE for *Q. ilex* or *Q. suber*. However, Gratani et al. (2003) and Ramírez-Valiente et al. (2010) did find differences among populations in this trait in the same species. These findings suggest that intraspecific variation in this trait might depend on the environmental conditions or how the populations were subsampled. Consequently, the possibility of intraspecific variation in WUE for *Q. oleoides* should not be ruled out.

### Relationships between leaf traits and growth rates across seasons

The present study showed strong relationships between photosynthetic rate per unit leaf area (A_area_), specific leaf area (SLA), and growth rates (AGR or RGR) (question iii). Photosynthetic rate per unit leaf area (A_area_) was positively associated with RGR in both wet and dry seasons (Table 4). A number of studies have shown that the maximum photosynthetic rate is positively associated with net assimilation rate (NAR), which represents the whole-plant carbon increase per unit leaf area and time (Poorter and Van der Werf, 1998). Although NAR is a balance between carbon gain in photosynthesis and carbon loss in respiration throughout a given period of time, light-saturated photosynthetic rate is a good predictor of NAR (Li et al., 2016). Traditionally, NAR has been considered to be only weakly related to RGR compared to other traits such as leaf mass ratio and specific leaf area or their product Leaf Area Ratio (Poorter and Remkes, 1990). Nevertheless, more recent studies have reported that net assimilation rates may be a primary determinant of RGR for specific functional groups, species or environments (Shipley, 2002, 2006; Villar et al., 2005). In oaks, A_area_ has been previously observed to be positively related to relative growth rate in temperate, Mediterranean and seasonally-dry tropical ecosystems (Cavender-Bares and Bazzaz, 2004; Castro-Díez et al., 2006; Ramírez-Valiente and Cavender-Bares, 2017). These observations suggest that on average, *Q. oleoides* seedlings with higher instantaneous carbon assimilation rates can sustain higher growth rates over longer periods of time particularly under favorable conditions of water.

Regardless of the season, specific leaf area (SLA) was negatively associated with RGR (Table 4, Table S2). These results are contrary to our hypothesis that LES traits would be positively associated with growth rates under favorable conditions. The growth benefits of low SLA have been usually explained in terms of reductions of water loss or higher water use efficiency in more sclerophyllous leaves (i.e., low SLA, high thickness) that allow higher potential to grow over longer periods of time when water is limiting during the growing season (Dudley, 1996; Lamont et al., 2002). Studies conducted with Mediterranean evergreen oaks agree with this previous evidence and have extensively reported negative associations between SLA and growth, particularly under severe drought conditions (Ramírez-Valiente et al., 2010, 2011, 2014, 2015a). Under well-watered conditions, positive relationships between SLA and growth rates are generally expected (Reich et al., 1997; Poorter and Bongers, 2006; Wright et al., 2010) although, studies with evergreen and deciduous oaks have also previously observed negative SLA-growth associations under well-watered conditions (e.g., Quero et al., 2008).

The unexpected negative SLA-growth relationship in *Quercus oleoides* may be mediated, at least in part, by the negative association observed between SLA and A_area_ (Table 5). In both seasons, SLA and A_area_ were negatively correlated, and A_area_ was positively correlated with RGR. The negative association between SLA and A_area_ contrasts expectations based on the worldwide leaf economic spectrum (LES) across species at the global scale (Reich et al., 1997; Wright et al., 2004), which shows that SLA is positively associated with mass-based photosynthesic rates (A_mass_) but not to area-based values (A_area_). In our study, SLA was positively associated with A_mass_ but more weakly than at the global scale (*r* = 0.44 in the wet season, *r* = 0.31 in the dry season, vs. *r* = 0.71 for Wright et al., 2004), whereas SLA was negatively related to A_area_ (*r* = −0.23 in the wet season and *r* = −0.14 in the dry season, vs. *r* = −0.05 for Wright et al., 2004). Recent studies have shown that global relationships between traits might not be applicable for specific group of species, biomes or environmental conditions (e.g., Edwards et al., 2014; Mason and Donovan, 2015; Grubb, 2016).

**Table 5:** **Pearson correlations among traits in dry (below diagonal) and wet (above diagonal) seasons**.

**Dry/wet**	**SLA**	**A_area_**	**A_mass_**	**g_s, area_**	**g_s, mass_**	**WUEi**	**Thickness^a^**	**${\text{N}}_{\text{mass}}^{\text{a}}$**	**δ^13^C^a^**
SLA		−**0.226**	**0.441**	0.013	**0.558**	−**0.268**			
A_area_	−**0.144**		**0.884**	**0.662**	**0.405**	**0.264**			
A_mass_	**0.306**	**0.757**		**0.607**	**0.746**	0.062			
g_s, area_	−0.023	**0.791**	**0.742**		**0.928**	−**0.516**			
g_s, mass_	**0.316**	**0.689**	**0.811**	**0.821**		−**0.585**			
WUEi	−**0.148**	−**0.176**	−**0.233**	−**0.675**	−**0.683**				
Thickness^a^	−**0.635**	**0.217**	−0.048	0.054	−**0.142**	0.141			
${\text{N}}_{\text{mass}}^{\text{a}}$	**0.299**	**0.333**	**0.460**	**0.205**	**0.306**	0.032	−**0.135**		
δ^13^C^a^	−0.054	**0.188**	**0.162**	0.003	−0.010	**0.147**	**0.296**	**0.357**	

*SLA, Specific Leaf Area; A_area_, area-based photosynthesis; A_mass_, mass-based photosynthesis; g_s, area_, area-based stomatal conductance; g_s, mass_, mass-based stomatal conductance; WUEi, intrinsic water use efficiency, Leaf thickness; N_mass_, mass-based nitrogen content; δ^13^C, 13-Carbon isotope composition (δ^13^C). Significant correlations (P < 0.05) are given in bold*.

a *Measured only in the dry season. Shaded area indicates combinations of traits for which correlations could not be performed*.

Interestingly, similar results have recently been found in other evergreen oaks. In particular, Niinemets (2015) observed a negative SLA-A_area_ relationship for *Quercus ilex* (*r* ≈ −0.40). He suggested that the negative SLA-A_area_ relationship could occur at the lower return end of the global LES spectrum due to increased mesophyll thickness. We measured leaf lamina thickness in the dry season and observed a strong negative relationship with SLA and positive relationships with A_area_, RGR, and AGR (Table 4). Similar results for SLA-thickness-A_area_-RGR relationships were found in our previous (greenhouse) study under both well-watered and dry treatments (Ramírez-Valiente and Cavender-Bares, 2017). Since leaf lamina thickness and mesophyll thickness are highly associated in oaks (e.g., Peguero-Pina et al., 2017), our results would support Niinemets (2015) hypothesis. “Multiple-trait” mixed models showed a significant effect of SLA on RGR even after taking A_area_ into account. The relationship between SLA and AGR, however, was weaker under dry conditions (significant *season by SLA* interaction in the AGR model), which agrees with the population-level trend showing a negative association between SLA and the index of moisture. However, the low R-coefficients observed in semi-partial correlations and the relatively weak population variation in SLA suggest that other unmeasured traits such as osmotic adjustment or biomass allocation patterns (Ramírez-Valiente and Cavender-Bares, 2017) might play a more important ecological role in *Q. oleoides*. In addition, survival during the earlier stages of the life cycle is known to be an important component of fitness in this species (Deacon and Cavender-Bares, 2015; Center et al., 2016), and future research needs to consider integrated measures of fitness (e.g., Shaw et al., 2008) to better understand trait-fitness relationships in *Quercus oleoides* seedlings.

In summary, our study revealed that xeric populations of tropical live oak exhibited higher growth rates (RGR and AGR) in the wet season but similar growth rates in the dry season. These findings are consistent with multi-species studies in seasonally-dry tropical ecosystems, which show that when dry seasons are long and wet seasons are warm species evolve toward resource-acquisitive strategies. Such species tend to have high carbon assimilation rates and fast growth rates to take advantage of favorable water availability conditions during the wet season. Our results also showed that *Quercus oleoides* individuals with leaves that have lower SLA and higher area-based photosynthetic rates had higher growth rates, particularly under well-watered conditions. This observation was partially explained by the negative association between specific leaf area (SLA) and area-based photosynthetic rates (A_area_), which is contrary LES expectations but similar to other recent intraspecific studies on evergreen oaks. Overall, our study shows the existence of important intraspecific genetic variation in leaf economics spectrum traits and growth rates in a long-lived tropical tree. These findings are critical to understanding how this keystone species will respond to climate change in the near future (Hällfors et al., 2016).

## Author contributions

JC-B conceived, designed and managed the common garden experiment with JRE and collected and transported seeds with AC. JC-B, AC, and JRE implemented the experiment. TL and GP maintained and oversaw the experiment and assisted in permit process. JPS and KLS performed the stable carbon isotope and leaf nitrogen analyses. JAR-V performed the gas exchange and leaf morphology measurements. JRE contributed to design the experiment and data collection. JAR-V analyzed the data. JAR-V wrote the manuscript with JC-B and revisions of all authors.

### Conflict of interest statement

The authors declare that the research was conducted in the absence of any commercial or financial relationships that could be construed as a potential conflict of interest.
