# Supplementary material for: Population-Level Differentiation in Growth Rates and Leaf Traits in Seedlings of the Neotropical Live Oak Quercus oleoides Grown under Natural and Manipulated Precipitation Regimes
Source: Front Plant Sci. 2017 May 9;8:585. doi: 10.3389/fpls.2017.00585 (PMC5423273; doi:10.3389/fpls.2017.00585)
Supplement: Supplementary file 1 [file DataSheet1.DOCX]

**Supplementary material**

Table S1. GLM results for intrinsic water use efficiency (WUEi). Factors: Season, Treatment, Region, covariate: stomatal conductance (g_s_)

|  | SS | df | *F* test | *P* |  |
| --- | --- | --- | --- | --- | --- |
| Season | 3365.3 | 1 | 40.42 | <0.001 | *** |
| Treatment | 755.7 | 3 | 3.03 | 0.036 | * |
| Region | 1215 | 3 | 4.86 | 0.004 | ** |
| g_s_ | 19738.6 | 2 | 118.53 | <0.001 | *** |
| Season × g_s_ | 177.3 | 2 | 1.06 | 0.351 |  |
| Treatment × g_s_ | 470.1 | 6 | 0.94 | 0.472 |  |
| Region × g_s_ | 176.8 | 6 | 0.35 | 0.905 |  |
| Season × Treatment | 1874.2 | 3 | 7.50 | <0.001 | *** |
| Season × Treatment × g_s_ | 608.3 | 6 | 1.22 | 0.309 |  |

**P* < 0.05, ***P* < 0.01, ****P* < 0.001. SS, sum of squares, df, degrees of freedom

Table S2. Multiple-trait mixed models results for RGR (relative growth rate) and AGR (absolute growth rate). Only traits that had a significant association with growth using Person correlations were included in the analysis. Mass-based photosynthetic rate and stomatal conductance were not included to avoid collinearity. Degrees of freedom (df), chi-square values (χ^2^) and significance *P* values were obtained from likelihood-ratio tests. **P* < 0.05, ***P* < 0.01, ****P* < 0.001.

|  |  | RGR | |  | AGR | |  |
| --- | --- | --- | --- | --- | --- | --- | --- |
|  | df | χ^2^ | *P* |  | χ^2^ | *P* |  |
| Population | 7 | 25.98 | 0.001 | *** | 15.26 | 0.033 | * |
| Season | 1 | 51.87 | < 0.001 | *** | 115.31 | < 0.001 | *** |
| Treatment | 3 | 5.11 | 0.164 |  | 3.99 | 0.263 |  |
| Population × Season | 7 | 24.89 | 0.001 | ** | 30.88 | < 0.001 | *** |
| Population × Treatment | 21 | 34.63 | 0.031 | * | 11.92 | 0.941 |  |
| Season × Treatment | 3 | 7.22 | 0.065 |  | 2.64 | 0.451 |  |
| Block (Treatment) | 1 | 22.06 | < 0.001 | *** | 10.84 | < 0.001 | *** |
| Family (Population) | 1 | 0 |  |  | 0 |  |  |
| Initial size | 1 | 217.05 | < 0.001 | *** | 76.68 | < 0.001 | *** |
| SLA | 1 | 39.68 | < 0.001 | *** | 17.63 | < 0.001 | *** |
| A_area_ | 1 | 29.08 | < 0.001 | *** | 15.70 | < 0.001 | *** |
| WUE | 1 | 0.04 | 0.838 |  | 0.40 | 0.527 |  |
| g_s,area_ | 1 | 4.70 | 0.030 | * | 2.47 | 0.116 |  |
| SLA × Season | 1 | 0.83 | 0.363 |  | 10.54 | 0.001 | *** |
| A_area_ × Season | 1 | 0.00 | 0.990 |  | 2.26 | 0.133 |  |
| WUEi × Season | 1 | 1.39 | 0.239 | . | 0.01 | 0.918 |  |
| g_s,area_ × Season | 1 | 0.21 | 0.647 |  | 1.70 | 0.192 |  |

SLA: Specific leaf area, WUEi: intrinsic water use efficiency A_area_: Area-based photosynthetic rate, g_s,area_: area-based stomatal conductance

Fig. S1. Relationship between area-based stomatal conductance (g_s,area_) and area-based photosynthetic rate (A_area_) for each combination of region, season and treatment. BZ: Belize (circles), HN: Honduras (triangles), DCR: Dry Costa Rica (diamonds), MCR: Mesic Costa Rica (squares). Dry season: red symbols, wet season: blue symbols. MCR, AMB and MCR, WDS could not be fitted to hyperboles. AMB: ambient, WWS: watering in the dry season, WDS: watering dry season, BOTH: watering in both seasons.

Fig S1. Continuation

Fig S1. Continuation

Fig S1. Continuation

Fig. S2. Watering treatment least-squared means (± SE) in the wet season (blue) and dry season (red) for specific leaf area (SLA) (a), area-based photosynthetic rate (A_area_) (b), mass-based photosynthetic rate (A_mass_) (c), area-based stomatal conductance (g_s,area_) (d), mass-based stomatal conductance (g_s,mass_) (e) and water use efficiency (WUEi) (f). AMB: ambient, WWS: watering in the dry season, WDS: watering dry season, BOTH: watering in both seasons. Only traits with *treatment* or *treatment by season* effects are shown.
